# Nutritional Composition, Antioxidant Activities, and Antiulcer Potential of *Lentinus squarrosulus* (Mont.) Mycelia Extract

**DOI:** 10.1155/2011/539356

**Published:** 2011-03-06

**Authors:** Nor Adila Mhd Omar, Noorlidah Abdullah, Umah Rani Kuppusamy, Mahmood Ameen Abdulla, Vikineswary Sabaratnam

**Affiliations:** ^1^Institute of Biological Sciences, Faculty of Science, University of Malaya, Kuala Lumpur 50603, Malaysia; ^2^Department of Molecular Medicine, Faculty of Medicine, University of Malaya, Kuala Lumpur 50603, Malaysia

## Abstract

Water extract of *Lentinus squarrosulus* mycelia was analysed for nutritional content, antioxidant capacity, and antiulcer ability. The extract contains high protein (57.6 g/100 g) and low total fat (0.5 g/100 g) and is rich in magnesium (0.4 g/100 g), potassium (3.8 g/100 g), vitamins B_1_ (1.42 mg/100 g), and B_3_ (194.29 mg/100 g) with total phenolic content of 39.16 mg/100 g. The cupric reducing antioxidant capacity and 1,1-diphenyl-2-picrylhydrazyl radical scavenging activity of the extract were A_450_ of 0.20 ± 0.03 at 0.5 mg/ml and IC_50_ of 14.29 mg/ml, respectively. Oral feeding of *L. squarrosulus* extract (250 mg/kg) offered significant gastric mucosal protection of Sprague-Dawley rats compared to cimetidine (50 mg/kg). The ulcer healing rate of ulcerated rats after 24, 48, and 72 hours of treatment was 82%, 90%, and 100%, respectively. The IL-1*β* level in the serum and the NF-*κ*B level in the tissues indicate that the healing potential was associated with attenuation of proinflammatory cytokines.

## 1. Introduction

Peptic ulcer disease is common, affecting millions of people yearly. The principal causes of peptic ulcer are infection by *Helicobacter pylori* and administration of NSAIDs (nonsteroidal anti-inflammatory drugs). *Helicobacter pylori* is the commonest cause of peptic ulceration, but only 15% of infected people develop an ulcer in their lifetime. Peptic ulcers developed due to an imbalance between aggressive factors (*H. pylori*, NSAIDs, gastric acid) and protective factors (mucin, bicarbonate, prostaglandins) leading to an interruption in the mucosal integrity [1]. NSAIDs are used worldwide for the treatment of pain, rheumatic, and cardiovascular diseases, and more recently, for the prevention of colon cancer and Alzheimer's disease [2]. Different NSAIDs confer different levels of risk, but even aspirin 75 mg/day, may occasionally cause serious ulceration [1]. It is known that stress, alcohol, and steroidal and nonsteroidal anti-inflammatory drugs are some of the factors that increased ulcer risk [3]. 

Most modern antiulcer drugs, which are currently available in the market, show limited efficacy against gastric diseases and are associated with severe side effects [4]. A drug with multiple mechanism of protective action including antioxidant activity may be highly effective in minimizing tissue injury in human diseases. It has been demonstrated that many drugs and formulations possess potent antioxidant action and are effective in healing experimentally induced gastric ulcers [5]. Reactive oxygen species (ROS), which cause tissue damage, are decreased by antioxidant enzymes such as endogenous glutathione (GSH), superoxide dismutase (SOD), glutathione S-transferase (GST), and catalase (CAT).

The ulcerated tissue damage is also accompanied by upregulation in nitric oxide synthase (NOS), epithelial cell apoptosis, and the induction of proinflammatory cytokines interleukin-1*β* (IL-1*β*) and tumor necrosis factor-alpha (TNF-*α*) that triggers nuclear factor-*κ*B (NF-*κ*B) activation. IL-1*β* has a wide spectrum of inflammatory, metabolic, and immunological properties. IL-1*β* plays a significant role in hippocampal synaptic function and is a potential genetic marker as indicator of gastric cancer risk. The prolongation of the healing was associated with an increase in gastric mucosal expression and the release of TNF-*α* and IL-1*β* [6]. It has been reported that NF-*κ*B plays an important role in gastric ulcer healing in rats. NF-*κ*B was activated in ulcerated tissue but not in normal mucosa, and the level of the activation was decreased with ulcer healing. The results demonstrate that NF-*κ*B activated in ulcerated tissue might upregulate the expression of healing-promoting factors responsible for gastric ulcer healing in rats [7]. 

Mushrooms have been part of the human diet for thousands of years. Most mushrooms are very important nutritionally and rich in protein, minerals, and vitamins. Mushrooms have been discovered to have therapeutic values, like anticancer, antitumoral, anti-cholesterol and antihemorrhagic effects. Most bioactive compounds that play essential roles in human and animal physiology have been found in many mushrooms. Okwulehie and Odunze reported that *Auricularia auricular judae*, *Pleurotus squarrosulus* and *Russula *sp. were found to contain appreciable amounts of alkaloids, phenols, saponins, and flavonoids [8]. *Hericium erinaceus* contains many biologically active compounds that have shown interesting biological activities, such as promotion of the synthesis of nerve growth factor, providing remedies for gastric ulcer and chronic gastricism, antitumor, antioxidant, and antimicrobial effects [8].

*Lentinus squarrosulus* is an edible mushroom commonly found in the wild and has not been cultivated on a large scale for the production of fruit bodies. The tough fruit body is rich in proteins, sugars, lipid, amino acids, vitamin B, C, and D, and minerals [9]. It has been reported that liquid fermentation of mushroom produces high amounts of uniform mycelial biomass as a source of bioactive compounds.

Mushroom mycelia have been reported to have high antioxidant properties. Hot water extract from *Agrocybe cylindracea* mycelia showed high 1,1-diphenyl-2-picrylhydrazyl (DPPH) radical scavenging ability and high reducing power [10]. *Antrodia cinnamomea* had potent antioxidant activity both *in vitro* and *in vivo* and showed protection of normal erythrocytes against oxidative damage [11]. Daker et al. also demonstrated that mycelia extract of *Marasmiellus* sp. possesses high antioxidant activity by the inhibition of lipid peroxidation [12].

Presently, there is no data available regarding the nutritional content, antioxidant capacity, and antiulcerogenic activity of *L. squarrosulus* mycelia extract. In this study, the antiulcer activity was assessed via prevention and treatment of gastric ulcers. The roles of proinflammatory cytokine, IL-1*β*, and the activation of NF-*κ*B in a model of ethanol-induced gastric ulcer in rats were also investigated.

## 2. Materials and Methods

### 2.1. Mushroom Mycelia

Mycelia of *L. squarrosulus* (KUM 50016) were obtained from Mycology Laboratory, Institute of Biological Sciences, University of Malaya and maintained on glucose (1.5%), yeast (0.8%), malt extract (0.8%), and peptone (0.8%) agar medium (GYMP). Seven days old *L. squarrosulus* mycelia grown on GYMP agar media at 25°C was used as inoculum. Five plugs cut from the periphery of the colony were transferred into 500 mL Erlenmeyer flasks containing sterile liquid GYMP media and incubated for two weeks at 25°C under static condition. 

### 2.2. Preparation of *Lentinus squarrosulus* Extract

The extract was obtained by water extraction of *L. squarrosulus* mycelial broth. Mycelia broth was homogenized in water at a ratio of 1 : 1 and boiled for 30 minutes. The broth was centrifuged at 3000 g for 15 minutes and the supernatant was filtered using Whatman no. 1 filter paper. The water extract was freeze-dried.

### 2.3. Nutritional Content of the Extract

Fifty grams sample of *L. squarrosulus* mycelia extract was analysed for nutritional components by Consolidated Laboratory (M) Sdn. Bhd.

### 2.4. In Vitro Antioxidant Capacity and Total Phenolic Content of the Extract

Antioxidant activity of *L. squarrosulus* extract was analyzed using DPPH, according to the method by Brand-Williams et al. [13]. Briefly, DPPH in methanol was prepared and 3.9 mL of this solution was added to 100 *μ*l extract dissolved in methanol at different concentrations (5, 10, 15, 20, and 25 mg/mL). The mixture was shaken vigorously, and the absorbance was measured at 515 nm. Lower absorbance of the reaction mixture indicated higher free radical scavenging activity which was expressed as IC_50._

The cupric reducing antioxidant capacity (CUPRAC) of the *L. squarrosulus *mycelia extract was determined according to the method of Apak et al., based on utilizing the copper (II)-neocuproine reagent as the chromogenic oxidizing agent [14]. The mixture of 1 mL of copper (II), neocuproine, and ammonium acetate buffer solution and extracts was added to make up a final volume of 4 mL. The absorbance at 450 nm was recorded against a reagent blank. The results of antioxidant activity were expressed in absorbance at 450 nm and compared with ascorbic acid as a positive control. 

Total phenolic content was measured using Folin-Ciocalteu method according to Singleton and Rossi and using gallic acid as a standard phenolic compound [15]. Briefly, 250 *μ*l of *L. squarrosulus* mycelia extract was added to 250 *μ*l of 10% Folin-Ciocalteu and incubated for 3 minutes. After 3 minutes, 500 *μ*l of 10% Na_2_CO_3_ was added and then allowed to stand for 1 hour in the dark. The absorbance was measured at 750 nm in a spectrophotometer. Phenolic content in the extract was expressed as gallic acid equivalents (GAE).

### 2.5. Antiulcer Potential of Extract

#### 2.5.1. Animals

Adult male and female Sprague-Dawley (SD) rats aged 6–8 weeks and weighed between 180 and 200 g were purchased from Animal Science Centre, University of Malaya, Kuala Lumpur, Malaysia. The animals were housed at 27 ± 2°C temperature, fed with standard laboratory pellet, and provided water *ad libitum*. Experimental protocols were approved by the ethical committee (which follows the guidelines of Animal Care and Use Committee), Laboratory Animal Science Centre, Faculty of Medicine, University of Malaya (Ethics no. ISB/11/02/2009/NAO(R)).

#### 2.5.2. Acute Toxicity Study

Acute toxicity (if any) of the extract was assessed based on the method by Cadirci et al. [16]. A total of 9 male and 9 female rats were divided randomly into 3 groups (*n* = 6), namely, control, low-dose, and high-dose groups. The rats were administered orally with *L. squarrosulus* mycelia extract at dose levels of 2 g/kg (low dose) and 5 g/kg (high dose) equivalent to a volume of 5 mL/kg body weight. Normal control rats received the same amount of vehicle (distilled water) only. Animals were observed carefully for 24 hours after extract administration and then for the next 14 days. At the end of this experimental period, the rats were observed for signs of toxicity, morphological behavior, and mortality. Acute toxicity was evaluated based on the number of deaths (if any).

#### 2.5.3. Ulcer Prevention Property

A total of 30 (15 males and females each) of SD rats were divided randomly into five groups of six rats in each group. All groups were deprived of food for 24 hours before the experiment. The experiment began with pretreatments according to the assigned group. Group 1 (ulcer control) received the vehicle (distilled water) only; Groups 2, 3, and 4 received 125, 250, and 500 mg/kg of extract, respectively, while Group 5 (positive control) received 50 mg/kg of cimetidine, an H_2_-receptor blocker. All animals were administered with absolute ethanol after thirty minutes of the pretreatment. After additional thirty minutes, all animals were sacrificed and their stomachs were removed and kept immersed in 10% of buffered formalin before the analysis of gastric lesions.

#### 2.5.4. Ulcer Healing Property

A total of 24 (12 males and females each) of SD rats were divided randomly into four groups comprising six rats in each group. Group 1 animals which served as normal control received vehicle (distilled water) only. The ulcerated groups (2, 3, and 4) were prefasted for 24 hours before inducing ulcer using absolute ethanol (5 mL/kg). Group 2 animals was treated with *L. squarrosulus* mycelia extract at a concentration of 250 mg/kg, group 3 were treated with 50 mg/kg cimetidine (positive control); and group 4 (non-treated group) were administered 5 mL/kg distilled water. All groups were treated once daily in the morning. One rat per group was sacrificed at 24, 48, and 72 hours after treatment to observe the ulcer index, and the blood was collected into commercial blood collection tubes (Z serum Sep Clot Activator) for analysis. The stomach tissues were homogenized with phosphate buffer saline (PBS). Both serum and tissue homogenates were stored at −80°C for analysis.

### 2.6. Gross Evaluation of Gastric Lesions

Each stomach was incised along a greater curvature and rinsed with tap water to remove gastric contents. The stomach was examined under a dissecting microscope (1.8x) with a square grid eyepiece (big square: length × width = 10 × 10 mm^2^ = ulcer area) to access the formation of ulcer area (hemorrhagic lesions). The sum of all lesions, in mm^2^, for each stomach was expressed as the ulcer area (mm^2^) [17]. The percentage of inhibition (%) was calculated by the following formula:

(1) $$\%\;\text{inhibition}=\left[\frac{{\text{UA}}_{\text{control}}-{\text{UA}}_{\text{treated}}}{{\text{UA}}_{\text{control}}}\right]\times 100.$$

### 2.7. Determination of IL-1*β*

IL-1*β* was determined by using AssayMax Human Interleukin-1*β* ELISA Kit (Catalog no. E12200-1, St. Charles, MO 63304). A murine monoclonal antibody specific for IL-1*β* has been precoated onto a microplate. IL-1*β* in standards and samples was sandwiched by the immobilized antibody and a biotinylated polyclonal antibody specific for IL-1*β*, which was recognized by a streptavidin-peroxidase conjugate. All unbound materials were then rinsed off, and a peroxidase enzyme substrate was added. The colour development was stopped, and the intensity of the color was measured immediately at a wavelength of 450 nm.

### 2.8. Determination of Total NF-*κ*B

The NF-*κ*B/p65 was measured by using the NF-*κ*B/p65 ActivELISA Kit (Catalog no. IMK-503, from IMGENEX Corporation, San Diego, CA 92121). The anti-p65-antibody-coated plate captures free p65 and the amount of bound p65 was detected by adding a second anti-p65 antibody followed by alkaline phosphatase- (AKP-) conjugated secondary antibody using colorimetric detection in an ELISA plate reader at a wavelength of 405 nm.

### 2.9. Statistical Analysis

The results were expressed as mean ± S.E.M. Statistical differences among groups were evaluated by Dunnett's multiple comparison test. Student's *t*-test was applied to comparisons between two groups. *P* values of <.05 were considered significant.

## 3. Results

### 3.1. Nutritional Content of the *L. squarrosulus* Extract

The nutritional components of *L. squarrosulus* mycelia extract are shown in Table 1. The crude extract contains high protein (57.6 g/100 g) and low total fat (0.5 g/100 g) and is rich in magnesium (0.4 g/100 g) and potassium (3.8 g/100 g) minerals, vitamins B_1 _(1.42 mg/100 g), and B_3_ (194.29 mg/100 g).

### 3.2. In Vitro Antioxidant Capacity of Extract

Table 2 shows the DPPH radical scavenging activity and cupric reducing power of *L. squarrosulus* mycelia extract compared to the positive control, ascorbic acid. DPPH radical was used as a stable free radical to determine antioxidant activity of natural compounds. Antioxidant activity was defined as the amount of antioxidant necessary to decrease the initial DPPH concentration by 50% (IC_50_; units = mg extract/mL methanol). The lower IC_50_ indicates the strongest ability of the extracts to act as DPPH scavengers. The IC_50_ of the extract was 14.29 mg/mL compared to ascorbic acid of 0.11 mg/mL. 

CUPRAC was estimated by the method described by Apak et al. [15]. Table 2 shows the absorbance of *L. squarrosulus* extract at 0.50 mg/mL (100 times higher concentration than positive control) was 0.20 ± 0.03, whereas ascorbic acid at 5 × 10^−3^ g/mL was 0.17 ± 0.04. 

Phenolics are important constituents with scavenging ability due to their hydroxyl groups and may contribute directly to the antioxidative action. The amount of total phenolic contents in *L. squarrosulus* mycelia extract was 39.16 mg/GAE g (Table 2).

### 3.3. Acute Toxicity Study

Rats that received oral doses of 2 g/kg and 5 g/kg did not manifest any clinical signs of toxicity. None of the doses tested could produce mortality in rats during the treatment period indicating that LD_50_, if any, should be higher than this dose. The present results showed that *L. squarrosulus* mycelia extract even at higher doses (2.5–5 g/kg) was well tolerated by rats.

### 3.4. Gross Evaluation of Gastric Lesions

#### 3.4.1. Prevention of Ulcer

**Figure 1:**
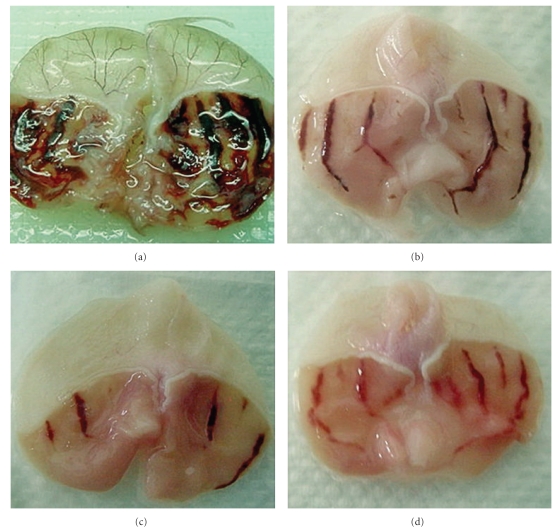
(a) Gross appearance of the gastric mucosa in a rat pretreated with 5 mL/kg of distilled water (ulcer control). Severe injuries are seen in the gastric mucosa. (b) Gross appearance of the gastric mucosa in a rat pre-treated with 5 mL/kg of *L. squarrosulus* mycelia extract (125 mg/kg). Injuries to the gastric mucosa are mild to moderate compared to the injuries seen in the ulcer control rat. (c) Gross appearance of the gastric mucosa in a rat pre-treated with 5 mL/kg of *L. squarrosulus* mycelia extract (250 mg/kg). Mild injuries to the gastric mucosa are seen and show flattening of gastric mucosa. (d) Gross appearance of the gastric mucosa in a pre-treated with 5 mL/kg of *L. squarrosulus* mycelia extract (500 mg/kg). Injuries of the gastric mucosa also show mild to moderate compared to the injuries seen in the ulcer control rat.

The groups orally pretreated with 50 mg/kg cimetidine, 125, 250, and 500 mg/kg of *L. squarrosulus* mycelia extract showed significantly (*P* < .05) reduced formation of gastric ulcers induced by ethanol. The inhibition percentage of ulcer area pretreated with 125, 250 and 500 mg/kg was 24%, 85%, and 18%, respectively, (Table 3, Figure 1). However, the best concentration of extract to prevent gastric ulcer was 250 mg/kg (Table 3, Figure 1(c)).

#### 3.4.2. Healing of Ulcer

Administration of 250 mg/kg of *L. squarrosulus* mycelia extract for 24, 48, and 72 days markedly accelerated the healing of gastric ulcer in ethanol-induced rats. *Lentinus squarrosulus* mycelia extract decreased the ulcer area by 82%, 90%, and 100% at 24, 48, and 72 hours of treatment, respectively, compared to cimetidine which shows a decrease in defective area in the ulcerated region by 60%, 82%, and 100%, respectively (Table 4).

#### 3.4.3. Determination of IL-1*β* Status in Serum

The concentration of IL-1*β* in serum after treatment with *L. squarrosulus* mycelia extract and untreated rats is depicted in Table 4. The concentrations of IL-1*β* were significantly increased after the administration of ethanol than the intact region of control rats. Treatment with extract attenuated these changes by decreasing the level of IL-1*β* after 72 hours of treatment (Table 4). The results suggest that there was an inhibition of neutrophil influx in the gastric mucosa caused by ulcerogen toxicity upon treatment with *L. squarrosulus* mycelia extract.

#### 3.4.4. Determination of NF-*κ*B in Gastric Tissues

The activation of NF-*κ*B level after 24, 48, and 72 hours of treatments is shown in Table 4. A significantly decreased activation of NF-*κ*B was observed between groups after 24, 48, and 72 hours of treatment.

## 4. Discussion

It has been reported that tropical mushrooms are rich in protein, minerals, and vitamins. Protein content of mushrooms is twice that of vegetables, four times that of oranges and significantly higher than that of wheat [18]. *Lentinus squarrosulus* mycelia extract studied possessed high protein (57.6 g/100 g) which contributes to 100% of RDA (50 g) and low total fat (0.5 g/100 g) and was rich in magnesium (0.4 g/100 g) which contributes to 100% of RDA (0.4 g) and potassium (3.8 g/100 g) which contributes to 100% of RDA (3.5 g), vitamin B_1_ (1.42 mg/100 g) which contributes almost 100% of RDA (1.5 mg), and vitamin B_3_ (194.29 g/100 g) which contributes to 100% of RDA (20 g). Mushrooms are quite high in protein (19–35%) including all the essential amino acids, low in fat, and are recommended as nutritional supplement for patients with cardiac problems. High level of potassium in mushroom extract suggests its utilization in antihypertensive diet in fact the consumption of fruits and vegetables higher in potassium can lower blood pressure. Mushrooms also contain relatively large amounts of carbohydrate and fiber, ranging from 51% to 88% and from 4% to 20% (dry weight), respectively, for the major cultivated species [19]. 

In this study, water extract of *L. squarrosulus* mycelia having total phenolic content of 39.16 mg/100 g GAE exhibited DPPH scavenging activity with IC_50_ value of 14.29 mg/mL, and CUPRAC value at 0.5 mg/mL was 0.20 ± 0.03 absorbance (Table 2). Cheung et al. reported that water extract of *Lentinus edodes* fruiting bodies showed the most potent radical scavenging activity, 55.4% in DPPH radical scavenging (at 6 mg/mL) compared to methanol extract [20]. According to Tsai et al., scavenging abilities of DPPH radicals were higher in *Agrocybe cylindracea* mycelia water extract, 1.66 mg/mL compared to extract of fruit bodies, 0.82 mg/mL [10]. Mycelia of *Marasmiellus *sp. fermented on maize were able to produce bioactive compounds with enhanced DPPH radical-scavenging ability, having IC_50_ value of 1.88 mg/mL [12]. Tsai et al. reported that the antioxidant activity from mycelia was higher than fruiting bodies of *Agrocybe cylindracea, G. tsugae*, *Lentinula edodes*, and *Pleurotus *spp. [10]. Previous study showed that the CUPRAC assay of stem root extracts of Rhubarb (*Rheum ribes*) at 50 *μ*g/mL was 1.25 [21]. 

Pihan et al. demonstrated that oxygen-derived free radicals are implicated in the mechanism of acute and chronic ulceration in the gastric mucosa, and scavenging these free radicals can play an appreciable role in healing these ulcers [22]. This study shows that *L. squarrosulus* mycelia extract significantly (*P* > .05) reduced the ulcer index and afforded significant protection against ethanol-induced ulcer. Ethanol causes vascular damage and necrosis on mucosa by increasing the release of vasoactive products from mast cells, macrophages, and other blood cells. Tissue damage begins with the formation of lipid radicals in cell membranes and continues with the conversion of these radicals to lipid hydroperoxides then finally to toxic products such as aldehydes, alkenes, and monoaldehydes [23]. It is feasible to speculate that the antioxidant potential of the extract could play an important role in the prevention and healing of gastric ulcer. 

 Macroscopic examinations showed the occurrence of stomach damage in all ethanol-induced gastric ulcer rats. Groups treated with *L. squarrosulus* mycelia extract and commercial drug (cimetidine) showed minor lesions. The gastric lesions were significantly reduced by the administration of *L. squarrosulus* mycelia extract at 125, 250, and 500 mg/kg (Figures 1(b), 1(c), and 1(d)). All doses of *L. squarrosulus* mycelia extract showed better gastroprotective effect in comparison to cimetidine, which is an H_2_-receptor blocker. However, the best concentration of extract to prevent gastric ulcer was 250 mg/kg. Damaged stomachs showed lesions in various forms and sizes dispersed on all stomach surfaces. Remarkable hyperemia and the surrounding blisters were observed in damaged stomachs. Hyperemia was at the highest degree in the ulcer control group. The severity of hyperemia paralleled with the extent of damage. As shown in Table 3, the percentage of the inhibition of ulcer treated with *L. squarrosulus* mycelia extract at concentration of 250 mg/kg was higher compared to other groups. 

*L. squarrosulus* mycelia extract at 250 mg/kg was selected to determine the ulcer healing property compared to cimetidine at 50 mg/mL. Oral administration of *L. squarrosulus* mycelia extract (250 mg/kg body weight/day) for 24, 48, and 72 hours significantly attenuated the biochemical changes caused by ethanol in rats. Percentages of inhibition of ulcer area in groups treated with cimetidine were lower compared to the extract. Ito et al. reported that cimetidine mainly accelerated the healing of gastric ulcers by the trophic action *via* the increase in gastric secretion [24]. This study suggested that the antioxidant property of *L. squarrosulus* mycelia extract contributes to the healing of ulcers.

An inflammation in gastric mucosa by an ethanol is accompanied by increased production of TNF-*α* which augments neutrophil-derived superoxide generation and stimulates the production of IL-1*β*, leading to neutrophil accumulation [25]. Both IL-1*β* and TNF-*α* are involved in the induction of inflammation, injury, and carcinogenesis in a variety of tissues including the gastric mucosa. In addition, IL-1*β* is considered as a major factor responsible for the induction of ulcer recurrence [26]. It has been reported that several plant constituents, including triterpenoids and polyphenolic compounds, are known to decrease TNF-*α* and IL-1*β* production [27]. The strong inhibitory action on serum IL-1*β* levels by *L. squarrosulus* mycelia extract may be due to triterpenoids and polyphenols present in the extract which is indicated by the high phenolic content (39.16 g/GAE g). These findings support the hypothesis that *L. squarrosulus* mycelia extract attenuates ethanol-induced neutrophil accumulation by inhibiting the production of proinflammatory cytokines. 

Takahashi et al. reported that NF-*κ*B was activated only in ulcerated tissue and that its activation is sustained during the healing of gastric ulcers in rats [7]. In resting cells, NF-*κ*B is coupled with inhibitor-*κ*B (I-*κ*B) and resides in the cytosol as an inactive form. In response to inflammatory stimuli mitogens, I-*κ*B is phosphorylated which then dissociates from NF-*κ*B. Subsequently, NF-*κ*B translocates into the nucleus to function as an active transcription factor. It has been speculated that NF-*κ*B is activated in ulcerated gastric tissue because gastric ulcers are associated with inflammation. Animal in groups treated with *L. squarrosulus* mycelia extract showed significantly lower level of NF-*κ*B compared to the untreated control group. 

Fungal metabolites were known to modulate the activity of NF-*κ*B [28]. It is well documented that fungi produce a large and varied number of biologically active compounds that not only stimulate the immune system but also modulate specific cellular responses by interfering with particular signal transduction pathway [29]. Panepoxydore, a compound found in *Lentinus crinitus*, interferes with the NF-*κ*B-mediated signal transduction by inhibiting the phosphorylation of NF-*κ*B. *Lentinula edodes* was shown to produce CAPE, which specifically inhibits the NF-*κ*B binding to DNA [30].

## 5. Conclusions

This study shows that *L. squarrosulus* mycelia extract can prevent ethanol-induced ulcers at a wide dose, range but a dose of 250 mg/kg of extract was the most effective. Toxicity studies of *L. squarrosulus* mycelia extract carried out in rats indicate no lethal effect at least up to an oral dose of 5 g/kg body weight indicating that LD_50_ of *L. squarrosulus* mycelia extract will be higher than this dose. Besides ulcer prevention, *L. squarrosulus* mycelia extract was also able to heal ulcer and this was associated with the attenuation of proinflammatory cytokines IL-1*β* and the inhibition of NF-*κ*B in ulcerated rats. Hence, *L. squarrosulus* mycelia extract being nontoxic and having a variety of nutritional value may potentially serve as nutraceutical ingredient for antiulcer prevention and treatment.

## Figures and Tables

**Table 1:** Nutritional analysis of *Lentinus squarrosulus *mycelia extract.

Test parameter	Method	Values	Recommended daily allowance (RDA)
Protein	Kjeldahl Method	57.6 g/100 g	50 g
Carbohydrate	By Calculation	12.4 g/100 g	300 g
Total fat	Solvent Extraction	0.5 g/100 g	65 g
Crude Fibre	AACC 32-10	<0.1 g/100 g	25 g
Selenium (as Se)	ICP-OES	<0.02 mg/kg	70 *μ*g
Iron (as Fe)	ICP-OES	2.17 mg/100 g	18 mg
Calcium (as Ca)	ICP-OES	32.04 mg/100 g	1000 mg
Magnesium (as Mg)	ICP-OES	0.4 g/100 g	0.4 g
Potassium (as K)	ICP-OES	3.8 g/100 g	3.5 g
Zinc (as Zn)	ICP-OES	2.85 mg/100 g	15 mg
Manganese (as Mn)	ICP-OES	0.17 mg/100 g	2.0 mg
Vitamin A	HPLC	<0.01 mg/100 g	5000 IU
Vitamin E	HPLC	<0.01 mg/100 g	30 IU
Vitamin B1	HPLC	1.42 mg/100 g	1.5 mg
Vitamin B2	HPLC	0.98 mg/100 g	1.7 mg
Vitamin B3	HPLC	194.29 mg/100 g	20 mg

Analysis of nutritional components in *L. squarrosulus* mycelia extract.

**Table 2:** The total phenolic contents and antioxidant capacity of *L. squarrosulus* extract compared with ascorbic acid.

	Total phenolic content GAE (mg/100 g)		DPPH (IC_50_ mg/mL)	CUPRAC	
				Concentration (*μ*g/mL)	(A_450_)
*L. squarrosulus* mycelia extract (0.05 *μ*g/mL)	39.16	*L. squarrosulus* mycelia extract	14.29	500	0.20 ± 0.03
Gallic acid (0.005 *μ*g/mL)	710.55	Ascorbic acid	0.11	5	0.17 ± 0.04

1,1-diphenyl-2-picryhydrazil radical (DPPH) results are expressed as inhibition concentration, (IC_50_). Cupric reducing antioxidant capacity (CUPRAC) results are expressed as mean ± standard deviation.

**Table 3:** Prevention of gastric ulcer by *L. squarrosulus* mycelia extract.

Treatment	Dose (mg/kg)	Ulcer area (mm^2^)	Inhibition (%)
Distilled water	—	841 ± 59^a^	—
Cimetidine	50	376 ± 25^b^	55
*L. squarrosulus* extract	125	640 ± 32^c^	24
	250	130 ± 13^d^	85
	500	686 ± 24^c^	18

All values were expressed as mean and ± standard error mean of six replicates of animals. Means with different superscripts was significantly different (*P* < .05), adjusted to the nearest mm.

**Table 4:** Healing of ethanol-induced ulcers by *L. squarrosulus* and the levels of IL-1*β* and NF-*κ*B after 24, 48, and 72 hours of treatment.

Treatment	Duration of treatment (hour)	Dose (mg/kg)	Ulcer area (mm^2^)	Inhibition (%)	IL-1*β* (pg/ml)	NF-*κ*B (ng/ml)
Without treatment	24	—	0 ± 0^a^	—	20.6 ± 1.2^a^	3.92 ± 0.71^a^
Distilled water		—	962 ± 11^b^	—	48.9 ± 2.7^b^	5.49 ± 0.52^a^
*L. squarrosulus* extract		250	172 ± 3^c^	82	65.8 ± 3.2^c^	4.51 ± 0.39^a^
Cimetidine		50	386 ± 3^d^	60	30.8 ± 1.5^d^	5.29 ± 0.68^a^

Without treatment	48	—	0 ± 0^a^	—	20.6 ± 1.2^a^	3.92 ± 0.71^a^
Distilled water		—	333 ± 7^b^	65	39.1 ± 0.7^b^	0.98 ± 0.20^b^
*L. squarrosulus* extract		250	95 ± 1^c^	90	47.2 ± 1.2^c^	0.78 ± 0.34^b^
Cimetidine		50	173 ± 16^d^	82	29.1 ± 0.8^d^	2.35 ± 0.00^ab^

Without treatment	72	—	0 ± 0^b^	—	20.6 ± 1.2^a^	3.92 ± 0.71^a^
Distilled water		—	82 ± 3^a^	92	37.8 ± 1.3^b^	0.78 ± 0.20^b^
*L. squarrosulus* extract		250	0 ± 0^b^	100	44.0 ± 1.3^c^	0.59 ± 0.00^b^
Cimetidine		50	0 ± 0^b^	100	28.2 ± 0.7^d^	1.57 ± 0.39^b^

The group “without treatment” refers to normal control group, “distilled water” refers to ulcer control group, “*L. squarrosulus* extract” refers to treated group and “cimetidine” refers to positive control group. All values were expressed as mean and ± standard error mean. Means with different superscripts were significantly different (*P* < .05), Six replicates animals were used. Concentration of extract tested was 250 mg/kg, while cimetidine was 50 mg/kg.
